# PRESCRIPTION DRUG USE FOR PAIN AND FOR SLEEP AND INCIDENT FRAILTY IN OLDER ADULTS

**DOI:** 10.1093/geroni/igz038.2509

**Published:** 2019-11-08

**Authors:** Gulcan Cil, Juyoung Park, Andrew W Bergen

**Affiliations:** 1 Oregon Research Institute, Eugene, Oregon, United States; 2 Florida Atlantic University, Boca Raton, Florida, United States

## Abstract

There is emerging evidence for association of polypharmacy with incident frailty. We performed a longitudinal study within the Health and Retirement Study (HRS) to address whether self-reported prescription drug use for pain and/or sleep (co-use or single use for pain or for sleep) influences incident frailty. We utilized data from the 2006–2014 waves of core and family member exit files in HRS to assign self-reported prescription drug use and sociodemographic and other drug use behavior variables as covariates and construct a Burden Model of frailty (≥ 0.2 ratio of positive/total indicators). We performed unadjusted and adjusted competing risk hazard model analysis with death as a competing risk. In a sample of 7,201 unique non-frail (at baseline) individuals (mean[SD] age 72[6.5] years, 54% female, 85% White, 12% African American, 7.3% Hispanic), prevalences of co-use and single-drug use for pain or for sleep were 2.2%, 14.9%, and 5.6%, respectively. Of 7,201 respondents, 2,723 (37.8%) became frail over the follow-up period and 713 (9.9%) died in non-frail state. The adjusted competing risk hazard model suggest that co-use and single use for pain or for sleep were associated with an increase in the risk of frailty by 92%, 58%, and 31%, respectively (p < .001), with statistically significant differences between all risk strata. Adjustment for baseline frailty score and selected chronic disease resulted in modest reductions in effect size with retention of significance. Validation of these initial findings should be undertaken with provider and pharmacy data to identify drug-, dosage-, and duration-specific risks.

